# Characterization of ceRNA network to reveal potential prognostic biomarkers in triple-negative breast cancer

**DOI:** 10.7717/peerj.7522

**Published:** 2019-09-09

**Authors:** Xiang Song, Chao Zhang, Zhaoyun Liu, Qi Liu, Kewen He, Zhiyong Yu

**Affiliations:** 1 School of Medicine and Life Sciences, University of Jinan-Shandong Academy of Medical Sciences, Jinan, Shandong, People’s Republic of China; 2 Department of Oncology, Shandong Cancer Hospital Affiliated to Shandong University, Shandong Academy of Medical Sciences, Jinan, Shandong, People’s Republic of China; 3 The People’s Hospital of Xintai City, Xintai, Shandong, People’s Republic of China; 4 School of Medicine, Shandong University, Jinan, Shandong, People’s Republic of China; 5 Department of Breast and Thyroid Surgery, Weifang Traditional Chinese Hospital, Weifang, Shandong, People’s Republic of China; 6 Department of Radiation Oncology, Shandong Cancer Hospital Affiliated to Shandong University, Shandong Academy of Medical Sciences, Jinan, Shandong, People’s Republic of China

**Keywords:** Triple-negative breast cancer, ceRNA network, Survival prognosis

## Abstract

Triple-negative breast cancer (TNBC) is a particular subtype of breast malignant tumor with poorer prognosis than other molecular subtypes. Previous studies have demonstrated that some abnormal expression of non-coding RNAs including microRNAs (miRNAs) and long non-coding RNAs (lncRNAs) were closely related to tumor cell proliferation, apoptosis, invasion, migration and drug sensitivity. However, the role of non-coding RNAs in the pathogenesis of TNBC is still unclear. In order to characterize the molecular mechanism of non-coding RNAs in TNBC, we downloaded RNA data and miRNA data from the cancer genome atlas database. We successfully identified 686 message RNAs (mRNAs), 26 miRNAs and 50 lncRNAs as key molecules for high risk of TNBC. Then, we hypothesized that the lncRNA–miRNA–mRNA regulatory axis positively correlates with TNBC and constructed a competitive endogenous RNA (ceRNA) network of TNBC. Our series of analyses has shown that five molecules (TERT, TRIML2, PHBP4, mir-1-3p, mir-133a-3p) were significantly associated with the prognosis of TNBC, and there is a prognostic ceRNA sub-network between those molecules. We mapped the Kaplan–Meier curve of RNA on the sub-network and also suggested that the expression level of the selected RNA is related to the survival rate of breast cancer. Reverse transcription-quantitative polymerase chain reaction showed that the expression level of TRIML2 in TNBC cells was higher than normal. In general, our findings have implications for predicting metastasis, predicting prognosis and discovering new therapeutic targets for TNBC.

## Introduction

The incidence rate of breast cancer ranks first among those of female malignant tumors (Ferlay et al., 2015; Siegel, Miller & Jemal, 2017). With the development of diagnosis and treatment methods, the incidence and mortality rates of breast cancer have been reduced annually, but the 10-year local recurrence rate of women under 40 years old is still 38% (Bollet et al., 2007). Triple-negative breast cancer (TNBC), as an important subtype of breast cancer, has stronger invasion, higher recurrence and metastasis rates, shorter survival time and more refractory treatment than those of non-TNBC due to negativities of estrogen receptor (ER), progesterone receptor (PR) and epidermal growth factor receptor 2 (HER2). Therefore, it is of great significance to clarify the molecular biological mechanisms for the onset and progression of TNBC for predicting metastasis, determining prognosis and developing innovative therapies.

Tumor biomarkers generally refer to characteristic indices for onset and progression, which can be objectively measured and evaluated to determine the tumor stage. Examining a disease-specific biomarker can help clinical identification, early diagnosis and prevention, as well as monitoring during treatment. In recent years, long non-coding RNA (lncRNA), microRNA (miRNA) and message RNA (mRNA) have been reported to play crucial roles in many biological processes, thus having become key biomarkers for the diagnosis and treatment of tumors (Hu et al., 2018; Lopez-Santillan et al., 2018; Markopoulos et al., 2018; Martens-Uzunova et al., 2014; Xing et al., 2015; Bollet et al., 2007). LncRNAs account for around 80% of all non-coding RNAs, with the lengths of over 200 nucleotides which are not capable of protein coding owing to the lack of meaningful open reading frames. Besides, miRNA refers to a single chain non-coding RNA molecules of 18–22 nt in length with high degree of conservatism, tissue specificity and spatial and temporal specificity. LncRNA can be a “molecular sponge” of miRNA, i.e., lncRNA binds miRNA to attenuate its silencing effects on target genes and to regulate them (Li et al., 2018). Therefore, the interactions between lncRNA and miRNA have been highlighted. LncRNA can compete with the target mRNA of miRNA to reduce free miRNA content and regulate such mRNA. Meanwhile, after binding to miRNA, lncRNA has become a target gene, and is destabilized through degradation. Finally, it is part of a complex RNA regulatory network.

Until now, the interaction mechanism for the regulatory network of lncRNA–miRNA–mRNA in TNBC remains unclear. Accordingly, we constructed a competitive endogenous RNA (ceRNA) topology network of breast cancer to screen prognosis-related RNA markers, and identified one processed pseudogene (PHBP4), two miRNAs (hsa-mir-133a-3p, hsa-mir-1-3p) and two mRNAs (TRIML2, TERT), with the expressions associated with survival and prognosis. The findings provide clues and ideas for further studying the molecular mechanisms of ceRNA in TNBC.

## Materials and Methods

### Data downloading and preprocessing

A total of 1,208 cases of BRCA patient’s RNA expression profile data (level3), and 1,080 relevant clinical data were obtained from the the cancer genome atlas (TCGA) database (https://www.cancer.gov/about-nci/organization/ccg/research/structural-genomics/tcga, data download time June 2018). mRNA and lncRNA expression data were selected for download in FPKM format. miRNA expression data was downloaded directly from the TCGA database on the Illumina HiSeq 2000 miRNA sequencing platform (Illumina Inc., San Diego, CA, USA). Since the TCGA database has normalized mRNA, lncRNA and miRNA expression data, no additional processing is required.

The criteria for screening data are as follows: (1) histopathological diagnosis is breast cancer; (2) screening for patients with loss of ER, PR and HER2 expression in immunohistochemistry according to the diagnostic criteria of TNBC; (3) pathology in patient information the staging and follow-up data should be complete; (4) the selected patients did not suffer from other types of malignant tumors. Based on the above screening criteria, we obtained RNA-seq data for 113 cases of paracancerous tissues and 112 samples of TNBC tissues (including 91 cases of stage I and II and 21 cases of stage III and IV). The miRNA-seq data were 104 cases of adjacent tissues, 79 cases of TNBC tissues (including 63 cases of Stage I and II, and 16 cases of stage III and IV). Subsequently, we screened differentially expressed RNAs at two levels, including early breast cancer tissue samples (stages I and II) vs. non-tumor, advanced ones (stages III and IV) vs. non-tumor.

Ethical approval was not in need, because the data were derived from a public database. The data were processed in accordance with TCGA guidelines (http://cancergenome.nih.gov/publications/publicationguidelines, last updated December 21, 2015). The two matrix files of RNA-seq and miRNA-seq with different samples were collected and annotated to the genome after being downloaded from the TCGA website. We then extracted both the expression spectra of mRNA and lncRNA from the RNA-seq matrix file, giving three matrix files of mRNA, lncRNA and miRNA expression spectra.

### Screening of differentially expressed genes

We employed the exact test function of the edge.R program from the R package for statistical analysis (Han et al., 2015). The edge.R language packet is mainly used to identify differentially expressed genes (DEGs) through the read numbers from different technical platforms (e.g., RNA-seq, SAGE and ChIP-seq). The obtained *p* values were corrected by FDR, and mRNA, lncRNA or miRNA with |logFC| > 3 and FDR < 0.001 was screened as DE-mRNA, DE-lncRNA or DE-miRNA. Subsequently, we screened differentially expressed RNAs by two sets of levels, including early breast cancer tissue samples (stages I and II) vs. non-tumor, advanced ones (stages III and IV) vs. non-tumor. Finally, we selected the intersection of lncRNAs, miRNAs and mRNAs for further bioinformatics analysis. We have drawn a flow chart to show the overall framework of the study (Fig. S1).

### Gene ontology and pathway enrichment analyses of DEGs

The above differentially expressed mRNAs were subjected to gene ontology (GO) analysis through the DAVID database (http://david.abcc.ncifcrf.gov/), and the significance of the above genes enriched in the Kyoto Encyclopedia of Genes and Genomes pathway was calculated by the hypergeometric test using the equation below:

$$p = 1 - \sum\limits_{k = 0}^m {{{(_k^n)(_{M - k}^{N - n})} \over {(_M^N)}}} $$

Where *N* is the number of genes in the whole genome, *M* is the number of genes annotated to a given pathway in the whole genome, *n* is the number of genes in the interaction network, and *m* is the number of genes annotated to a given pathway. *p* < 0.05 was set as the cut-off criterion.

### Construction of ceRNA regulatory network

The lncRNAs–miRNAs–mRNAs regulatory network was obtained based on the regulatory pairs of lncRNAs–miRNAs and miRNAs–mRNAs. If the correlation coefficient (*r*) between DE-RNA and DE-miRNA is <−0.3 and *p* < 0.05, they are considered to be negative regulatory co-expression and then predicted by mircode (http://www.mircode.org) for the regulatory relationship between DE-lncRNA and DE-miRNA. The regulatory relationships between DE-miRNAs and DE-mRNAs were predicted by TargetScan (http://www.targetscan.org) (Sarver & Subramanian, 2012). DE-miRNAs and DE-mRNAs pairs as well as DE-lncRNAs and DE-miRNAs pairs with opposite expression trends were selected to construct networks, giving DE-mRNAs and DE-lncRNAs regulated by the same DE-miRNA. Cytoscape software (3.5.1 version) was used to further visualize the co-expression network of lncRNA–miRNAs–mRNA (Van Parys et al., 2017).

### Pathway and GO analysis of differentially expressed mRNAs in ceRNA network and construction of protein–protein interaction network

Genes in the above networks were extracted, and GO term and pathway enrichment analyses were performed for the common DEGs by Metascape database. Protein–protein interaction (PPI) network analysis (score >0.4) was conducted by String database version10.5. MCODE is a plugin for Cytoscape for filtering modules from a PPI network. Provides a module with an MCODE score >3 and a number of nodes >3. A set of miRNAs, including the miR-200 and miR-182/183 family members, co-operate in post-transcriptional regulation, both reinforcing and buffering transcriptional output (Cursons et al., 2018).

### Prognostic analysis of ceRNA network

The survival data of each sample was extracted from the clinical information of the above sample downloaded from TCGA database. Univariate Cox proportional hazards regression and Kaplan–Meier (K–M) survival analysis were performed to determine the relationship between lncRNAs/miRNAs/mRNAs in ceRNA networks and survival time. The Log rank method was used to analyze the relationship between RNA expression and survival time (*p* < 0.05 was considered statistically significant). The K–M survival curve was plotted by using R package survival for each node in the constructed ceRNA topology network to analyze the survival differences among mRNA, lncRNA and miRNA in the ceRNA module. Survival was analyzed by the K–M method and the log-rank test. *p* < 0.05 was considered statistically significant.

### Cell culture

Human normal mammary cell line HTB-125 (HS578BST), together with TNBC cell lines MDA-MB-231 and MDA-MB-468 were obtained from China Center for Type Culture Collection, Chinese Academy of Sciences (Shanghai, China). All cell lines were grown in the Dulbecco’s modified Eagle medium (HyClone; GE Healthcare Life Sciences, Logan, UT, USA) containing 10% fetal bovine serum and penicillin/streptomycin (Thermo Fisher Scientific Inc., Waltham, MA, USA). All cell lines were cultured in a 37 °C incubator with 5% CO_2_.

### RNA extraction and reverse transcription-quantitative polymerase chain reaction

According to the manufacturer’s protocol, total RNA was extracted from Human normal mammary cell line HTB-125 (HS578BST), TNBC cell lines MDA-MB-231 and MDA-MB-468 by TRIzol reagent (Thermo Fisher Scientific Inc., Waltham, MA, USA). RevertAid first strand cDNA synthesis kit (Fermentas; Thermo Fisher Scientific Inc., Waltham, MA, USA) was employed to generate cDNA. SYBR-Green qPCR mixture (Toyobo Co., Ltd., Osaka, Japan) was used to carry out qPCR with Thermal Cycler Dice Real-Time System III TP950 1 Set (Takara Bio, Inc., Otsu, Japan). GAPDH (for mRNA) was utilized as an internal control. Primer sequences for qPCR: Forward, GCCACCGAGCTAGAGGAGAT; Reverse, CTTGAGCAATGCCAAGGTGC. Thermal cycling conditions for qPCR: 95 °C for 5 min, followed by 40 cycles of denaturation at 95 °C for 15 s and annealing/extension at 60 °C for 30 s. The relative expression of each gene was calculated by 2−ΔΔCq. All tests were repeated at least three times.

## Results

### Screening of DEGs

**Figure 1:**
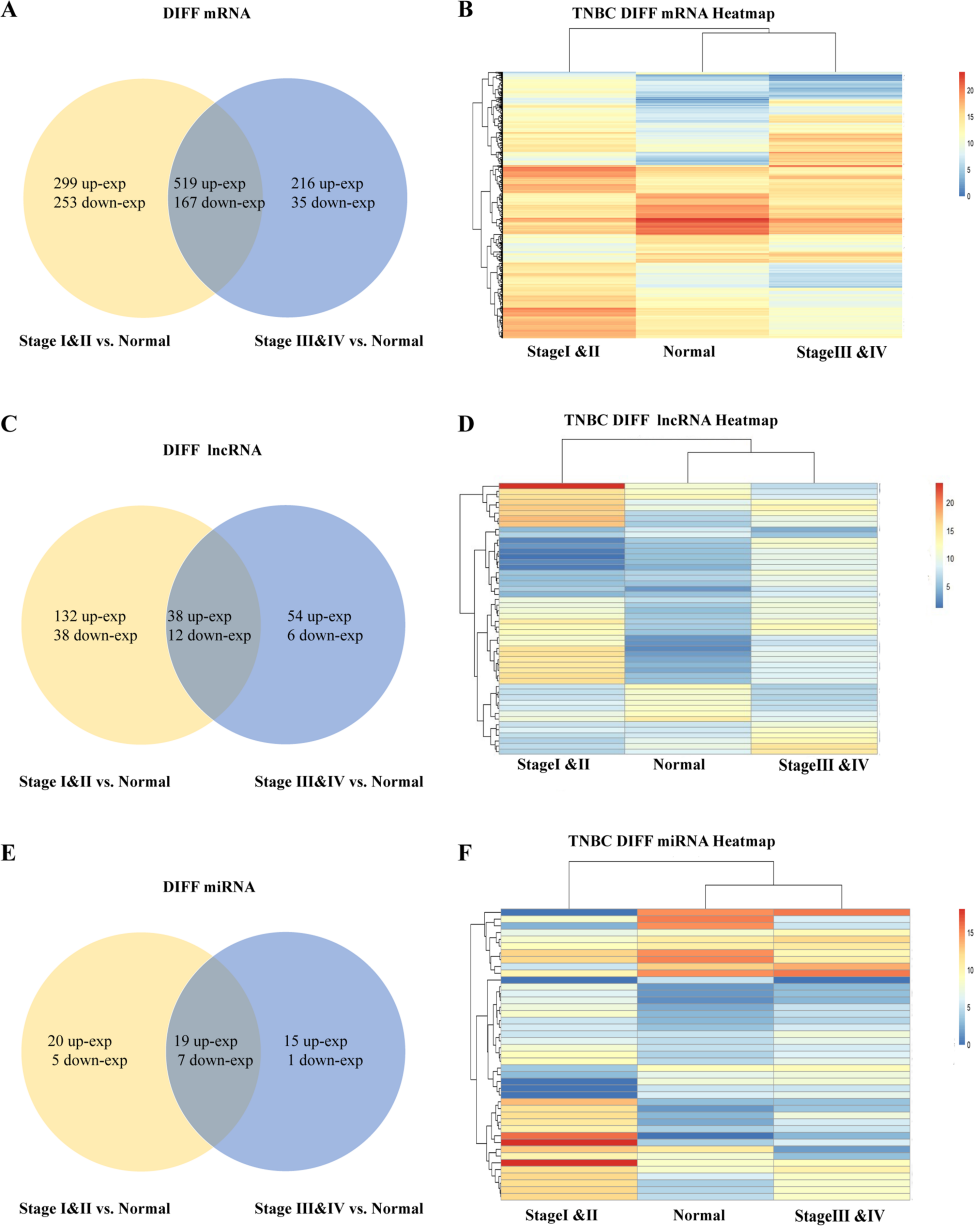
Venn diagrams and Heat maps. (A, C, E) Venn diagrams represent the differentially expressed mRNAs, lncRNAs, miRNAs obtained from the TCGA data among stages III–IV and stages I–II in triple-negative breast cancer, respectively. (B, D, F) The heatmap of differentially expressed mRNAs, lncRNAs, miRNAs between triple-negative breast cancer groups and controls. Stages I–II represents the earlystage group, stages III–IV represents late-stage group, respectively. The control (N) represents adjacent non-tumor tissues.

Differentially expressed RNAs between TNBC and control groups were analyzed. Using |logFC| > 3, FDR < 0.001 as a screening criterion for differential expression of mRNAs, lncRNAs, and miRNAs. There were 1,238 (including 818 up, 420 down) and 220 (including 170 up and 50 down) differentially expressed mRNAs and lncRNAs between early and control groups, respectively, and 937 (including 735 up, 202 down) and 110 (including 92 up and 18 down) differentially expressed mRNAs and lncRNAs between advanced and control groups, respectively. In addition, there were 51 (including 39 up and 12 down) differentially expressed miRNAs between early and control groups, and 42 (including 34 up, 8 down) ones between advanced and control groups. Information about these DE-mRNAs, DE-lncRNAs, DE-miRNAs were placed in Supplemental Material 1. The heat maps and Venn diagrams were plotted to illustrate the common characteristics of gene expressions among different groups or the unique gene distribution of each breast cancer group. Compared with the control group, a total of 686 common differentially expressed mRNAs were found in early and advanced groups, of which 519 were up-regulated and 167 were down-regulated (Figs. 1A and 1B). There were 50 differentially expressed lncRNAs, of which 38 were up-regulated and 12 were down-regulated (Figs. 1C and 1D). Additionally, there were 26 differentially expressed miRNAs, of which 19 were up-regulated and seven were down-regulated (Figs. 1E and 1F).

### GO and pathway enrichment analyses of DEGs

**Figure 2:**
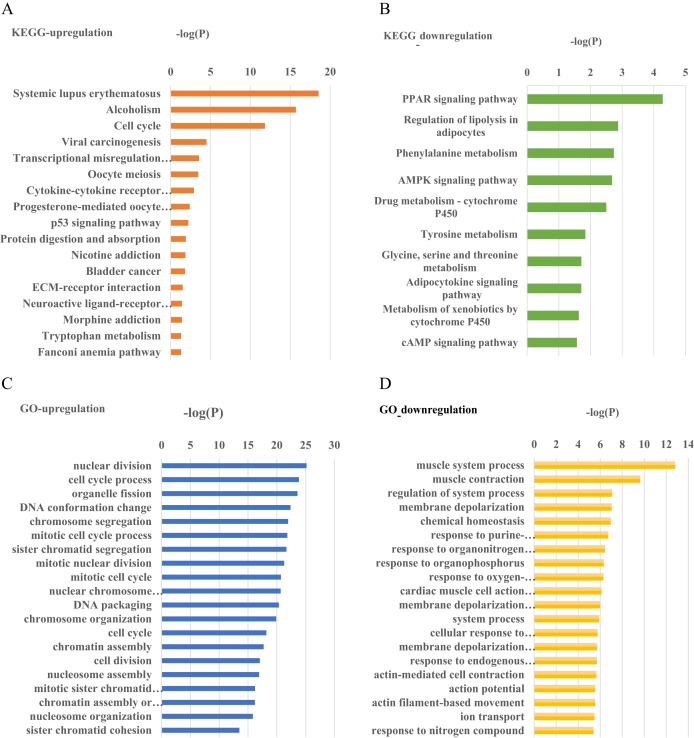
GO and pathway enrichment analysis histograms of differentially expressed mRNAs. (A, B) Pathway enrichment analysis histograms of up- and down-regulated genes; (C, D) GO enrichment analysis histograms of up- and down-regulated genes.

We employed the DAVID database (http://david.abcc.ncifcrf.gov/) to conduct GO and pathway analyses for the above differentially expressed mRNAs (Fig. 2). Detailed results of the GO and pathway analysis are attached to Tables S1–S4. The up-regulated genes were involved (identified) in the signaling pathways including cell cycle, p53 and ECM-receptor interaction (Fig. 2A), and the down-regulated genes were involved including the cAMP and AMPK signaling pathways (Fig. 2B). It is considered that there is enrichment in the GO terms and pathways if *p*-value is <0.05.

### Construction of ceRNA regulatory network

**Figure 3:**
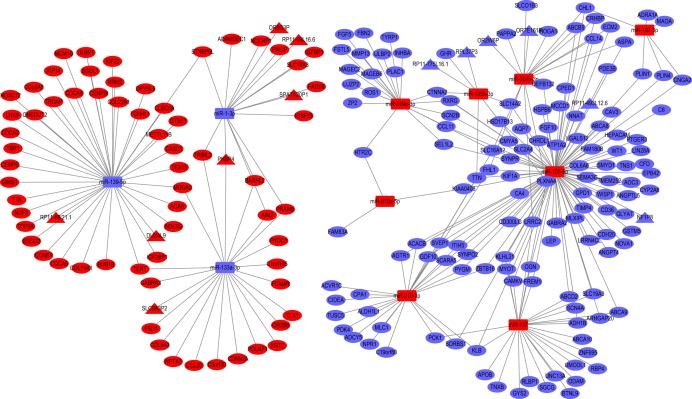
The miRNA–lncRNA–mRNA ceRNA network. Red round rectangle represent up-regulated miRNAs, while blue round rectangle represent down-regulated miRNAs. Triangles and circles represent lncRNAs and mRNAs, respectively, while red indicates up-regulation, and blue indicates down-regulation.

Because ceRNA network is mainly the regulatory mode of “lncRNA–miRNA–mRNA.” The mRNA and lncRNA were linked with same miRNA. Thus “lncRNA–miRNA–mRNA” forming ceRNA network. So, miRNA is the key core of lncRNA and mRNA regulating network. In this study, the correlation coefficient between RNA (lncRNA or mRNA) and miRNA is *r* < −0.3 with *p* < 0.05, as a screening criterion that may form a regulatory relationship of ceRNA. Subsequently, the relationships between DE-mRNAs and DE-miRNAs conforming to the above criteria were compared in the TargetScan database, and the relationships between DE-lncRNAs and DE-miRNAs were compared in the miRanda database. Finally, the aligned DE-mRNA and DE-miRNA pairs, and the DE-lncRNA and DE-miRNA, construct a ceRNA network using the shared DE-miRNA as a junction. Based on the analyses above, 262 miRNA–mRNA pairs and 14 lncRNA–miRNA pairs were obtained (Table S5). Subsequently, we combined the files of these pairs and visualized them using Cytoscape, yielding a ceRNA topology network (Fig. 3) (Cheng, An & Li, 2017).

### Pathway and GO analyses of differentially expressed mRNAs in ceRNA network and construction of PPI network

**Figure 4:**
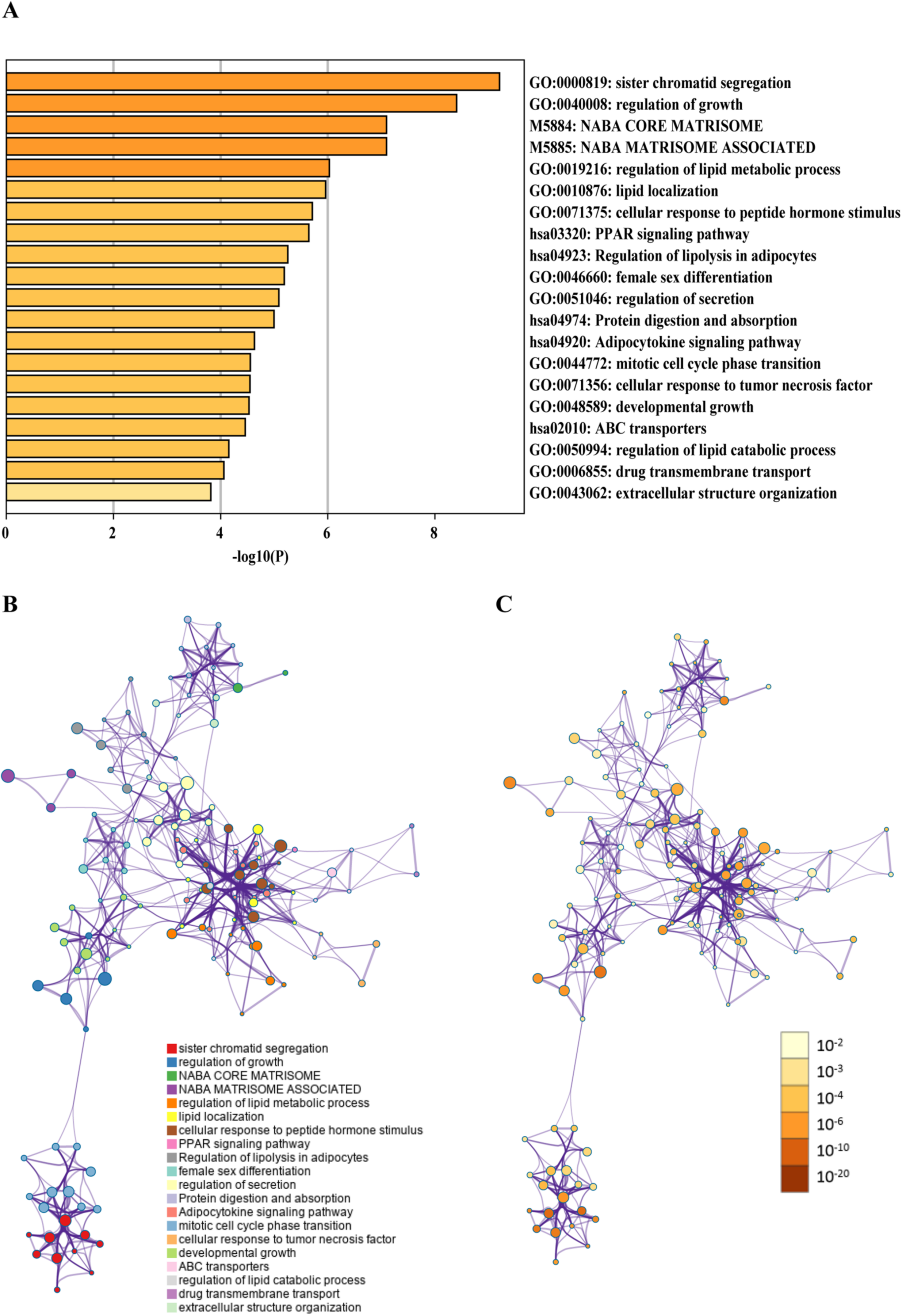
Integrative analysis of differentially expressed mRNAs in ceRNA network by metascape. (A) Heat map representing the top 20 significantly enriched functions and pathways; (B) network showing function and pathway correlations. Different colors in the map represented different function groups. (C) Network of functional and pathway enrichment. The darker the color, the more genes that are enriched in this pathway or biological process.

Gene ontology term and pathway enrichment analyses were performed for differentially expressed mRNAs using the Metascape database (Tian et al., 2018), and the top 20 significant pathways and functions were selected in accordance with *p* values to plot heat maps (Fig. 4). Raw data from the metascape analysis was placed in Supplemental Material 2. GO analysis showed that regulation of growth, regulation of secretion and mitotic cell cycle phase transition were significantly enriched biological processes, and the signaling pathways of PPAR, protein digestion and absorption, and regulation of lipolysis in adipocytes were involved. Different colors in Figs. 4B and 4C represent various categories, based on the enrichment degree of pathway or function. A darker color means that more genes are enriched in this pathway or biological process.

**Figure 5:**
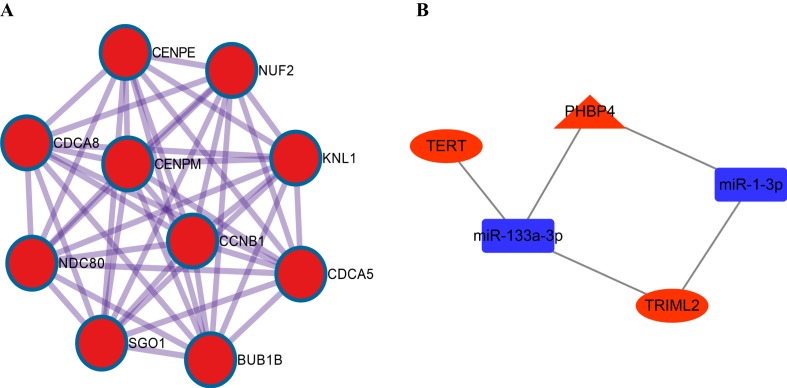
PPI network and ceRNA sub-network. (A) Each node represents a protein, and the edge between nodes represents the interaction between two connected proteins. (B) Prognosis-related ceRNA sub-network, where red round rectangle indicates miRNA, riangles and circles represent lncRNAs and mRNAs, respectively.

To further explore the interaction of DE-mRNA in the ceRNA network, we conducted PPI network analysis (Fig. S2; Table S6) from the String database version 10.5. In addition, based on the PPI network, module analysis is performed through the MCODE plug-in to screen out a top enriched module (Fig. 5A). However, the resulting module was not correlated with the survival-related genes screened out.

### Prognostic analysis of ceRNA network nodes

**Figure 6:**
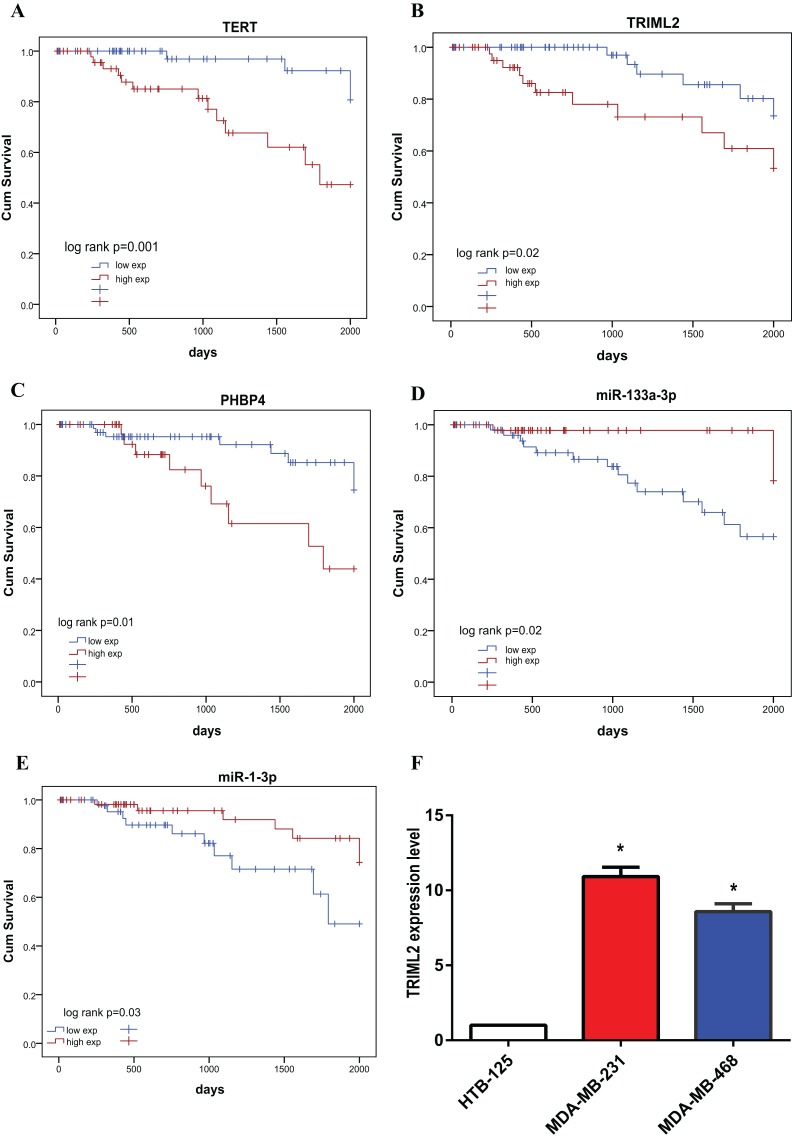
Survival curves of prognosis-related genes in ceRNAs. Expressions of TRIML2 in normal mammary and TNBC cells. Horizontal axis: overall survival time, days; vertical axis: survival function. (A) Relationship between TERT expression and overall survival of patients with triple-negative breast cancer; (B) relationship between TRIML2 expression and overall survival of patients with triple-negative breast cancer; (C) relationship between PHBP4 expression and overall survival of patients with triple-negative breast cancer; (D) relationship between hsa-mir-133a-3p expression and overall survival of patients with triple-negative breast cancer; (E) relationship between hsa-mir-1-3p expression and overall survival of patients with triple-negative breast cancer; (F) differences between expression levels of TRIML2 in human normal mammary and TNBC cell lines. *indicates that the *p* value < 0.05

We subjected 14 lncRNAs, 11 miRNAs and 262 mRNAs in the network to K–M survival (Survminer Software package) analysis, respectively. Finally, we selected five molecules that were significantly correlated with prognosis (i.e., TERT, TRIML2, PHBP4, hsa-mir-1-3p and hsa-mir-133a-3p) to construct a prognostic ceRNA sub-network (Fig. 5B). The corresponding K–M survival curves are exhibited in Fig. 6. We then analyzed TRIML2 gene in the further study.

### Expressions of TRIML2 in normal mammary and TNBC cells

Reverse transcription-quantitative polymerase chain reaction (RT-qPCR) showed that the expression levels of TRIML2 in TNBC cell lines were significantly higher than that in normal mammary cell line (Fig. 6F).

## Discussion

Breast cancer is the most prevalent and lethal malignancy among women in the world, with highly heterogeneous biological and clinical characteristics. Molecular biological studies on TNBC are of great significance to the survival of patients with specific genetic backgrounds (Kuo & Caughey, 2018; Lyman et al., 2018). In recent years, the application of high-throughput technology and advances in bioinformatics technology has enhanced our understanding of the molecular level of breast cancer. In this study, transcriptome sequencing data and clinically relevant pathological features of breast cancer were obtained from the TCGA database. After integration analysis, we constructed a ceRNA topology network, and screened and identified five RNA markers that may be related to TNBC prognosis: hsa-mir-133a-3p, hsa-mir-1-3p, TRIML2, TERT and PHBP4.

P53 is a tumor suppressor gene, and interference of the p53 signaling pathway is involved in the onset and progression of many cancers. Restoring its functions has significant inhibitory effects on tumors (Dastjerdi et al., 2016; Ohara et al., 2016). One of the mRNAs that we screened from the prognostic ceRNA network was TRIML2, as a TRIM protein family member capable of trans-activation and extension of p53 (Kung et al., 2015), TRIML2 can increase the p53 protein level through small ubiquitin-like modifier. Additionally, the mutation rate of TP53 in TNBC is above 80%. TRIML2 is a target of TP53, the deregulation of which may be attributed to the mutation of TP53, further affecting the regulation of certain cell biology processes.

TRIM family proteins affect cell growth, differentiation, proliferation, apoptosis and other tumor-related biological processes (Carvajal & Manfredi, 2013; Vousden & Lu, 2002). In this study, TCGA data analysis showed that TRIML2 expression in TNBC tissues was significantly higher than that in adjacent tissues. As evidenced by RT-qPCR, its expression levels in TNBC cells also exceeded that of normal mammary cells. We also show that TRIML2 is significantly related to the survival in TNBC. Moreover, we screened TERT which is the catalytic subunit and one of the core components of telomerase, also as the most important rate-limiting factor for telomerase to function (Spilsbury et al., 2015). As cells mature, the activity of telomerase gradually decreases, and the telomere gradually shortens until chromosomes are degraded, which then causes cell death. It is well-documented that TERT was highly expressed in different cancers, with its promoter mutation being a possible marker for the poor diagnosis and prognosis of urothelial, bladder, thyroid papillary, prostate cancers, etc. (Borah et al., 2015; Jaiswal et al., 2017; Liu et al., 2014; Nickerson et al., 2014; Stoehr et al., 2015).

As is known to all, miRNAs are important post-transcriptional regulators of gene expression. Each miRNA can regulate the expressions of tens or even hundreds of target mRNAs, dominantly participating in cell proliferation, metastasis, differentiation, development, apoptosis and other biological processes (Kawano & Nakamachi, 2011; Ma et al., 2016; Xie & Sadovsky, 2016). We screened two miRNAs (hsa-mir-133a-3p, hsa-mir-1-3p) through the ceRNA topology network. As the first officially numbered human miRNA, mir-1-3p can significantly inhibit the proliferation, invasion, migration and other biological processes of prostate cancer, hepatocellular carcinoma, gallbladder cancer, non-small cell lung cancer, gastric cancer, etc. (Han et al., 2015; Karatas et al., 2014; Letelier et al., 2014; Li et al., 2012; Zhao et al., 2014). Likewise, mir-133a-3p has also been verified as a tumor suppressor gene in colorectal cancer, ovarian cancer, breast cancer, bladder cancer and so on (Cui et al., 2013; Dong et al., 2013; Kojima et al., 2012).

Prohibitin pseudogene 4 (PHBP4) has seldom been studied hitherto, requiring in-depth research on its unknown biological functions. This gene is about one kb with its ORF <30–150 bp and could be encode a lncRNA. Pseudogene RNA is a kind of lncRNA. Some pseudogenes could function to buffer miRNA mediated RNAi to enable the true gene mRNA translation. That may be one of the mechanisms by which PHBP4 works (Sato et al., 1993).

In this study, we performed GO and pathway analyses for differentially expressed mRNAs predicted from the database. These DEGs were significantly enriched in cancer-related pathways, such as p53, cAMP and AMPK signaling pathways. As described above, p53 gene plays major roles in many biological pathways, including cell cycle, growth, differentiation, apoptosis and death. Similarly, the cAMP signaling pathway also predominantly participates in cellular processes such as immune function, growth, differentiation and metabolism (Serezani et al., 2008). Furthermore, AMPK enzyme, as a negative regulator of glycolysis, inhibits tumor progression by regulating a part of the proliferative and metabolic pathways (Sanli et al., 2014). In addition, the K–M survival curves plotted for RNAs from the prognostic ceRNA sub-network revealed that the RNA expression levels were significantly correlated with the survival rate of patients with TNBC.

There were some limits in this study. Firstly, we hope to further explore the interaction of prognosis-related molecules by establishing a PPI interaction network for mRNA in ceRNA networks. Unfortunately, neither PPI network nor MCODE analysis showed interaction of TRIML2 and TERT with other molecules. Secondly, TRIML2 was verified by qPCR and its high expression in TNBC cell line was confirmed. However, the expression of TRIML2 in cell lines of other subtypes of breast cancer was not detected. In this study, we identified molecules that may be important for TNBC, but such a study can offer no further level of details definitively. In addition, the supporting data is very limited, and more targets are proposed as competing targets in the network should be studied in the future.

## Conclusion

In summary, we successfully screened specific lncRNAs, miRNAs and mRNAs in TNBC from the TCGA database, and established a ceRNA topology network from which we selected and further studied five specific RNAs: hsa-mir-133a-3p, hsa-mir-1-3p, TRIML2, TERT and PHBP4. Moreover, we conducted RT-qPCR to further validate these results. The expression levels of TRIML2 gene in TNBC tissues and cells surpassed those in normal paracancerous tissues and mammary cells. The findings provide potentially eligible targets and ideas for the future diagnosis and treatment of TNBC.

## Supplemental Information

10.7717/peerj.7522/supp-1 Supplemental Information 1 Flow chart of the ceRNA network construction.

10.7717/peerj.7522/supp-2 Supplemental Information 2 The PPI network from the String database. Each node represents a protein, and the edge between nodes represents the interaction between two connected proteins.

10.7717/peerj.7522/supp-3 Supplemental Information 3 Information about DE-mRNA, DE-lncRNA, DE-miRNA.

10.7717/peerj.7522/supp-4 Supplemental Information 4 Raw data about metascape analysis.

10.7717/peerj.7522/supp-5 Supplemental Information 5 Detailed results of the GO and pathway analysis.

10.7717/peerj.7522/supp-6 Supplemental Information 6 ceRNA network data.

10.7717/peerj.7522/supp-7 Supplemental Information 7 PPI network from String database.

## Additional Information and Declarations

### Data Availability

The following information was supplied regarding data availability: All data is available as Supplemental Files.
