# Supplementary figures and images for: Characterization of ceRNA network to reveal potential prognostic biomarkers in triple-negative breast cancer
Source: PeerJ. 2019 Sep 9;7:e7522. doi: 10.7717/peerj.7522 (PMC6741283; doi:10.7717/peerj.7522)

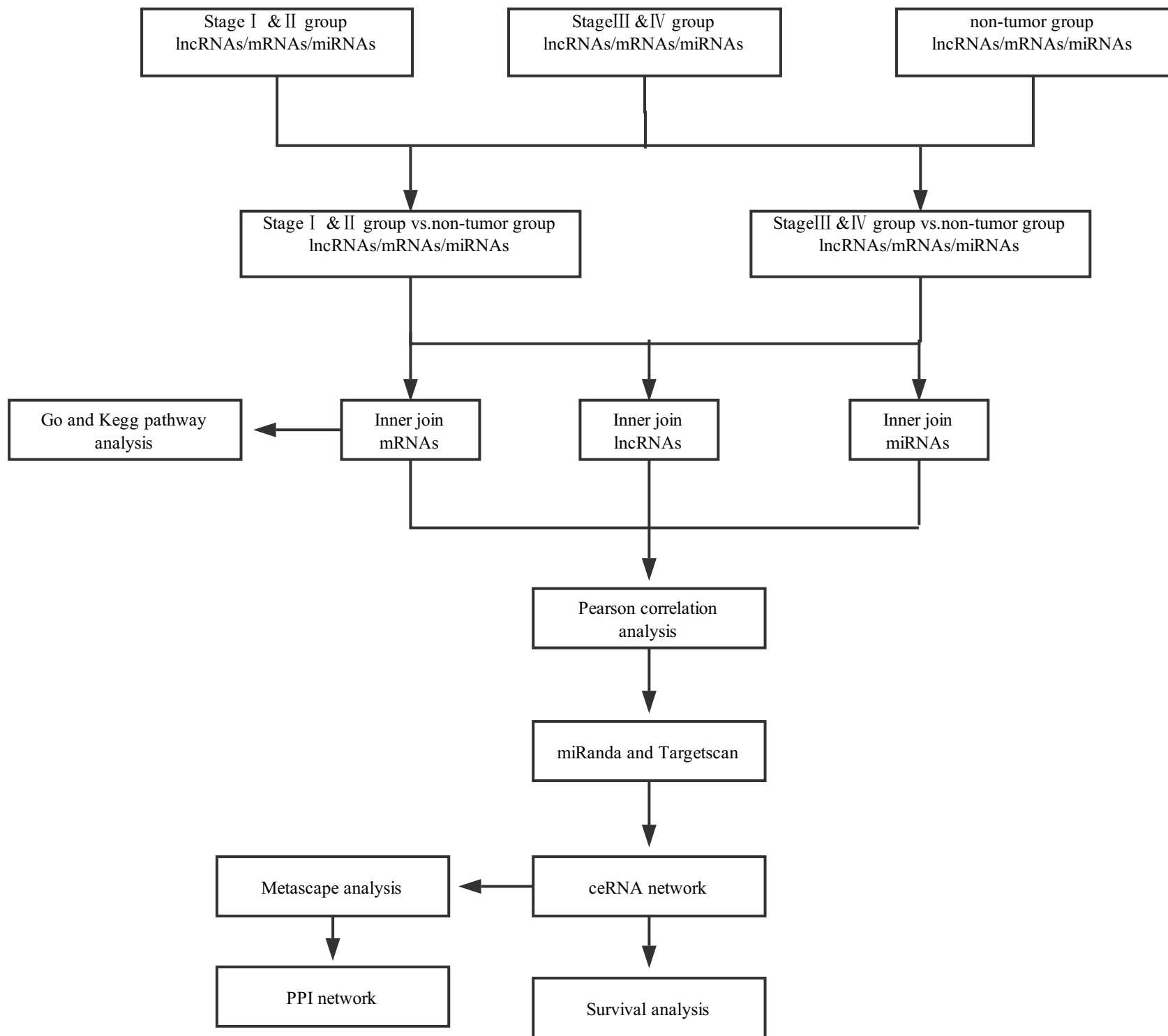

Supplement: Supplemental Information 1 [file peerj-07-7522-s001.pdf]

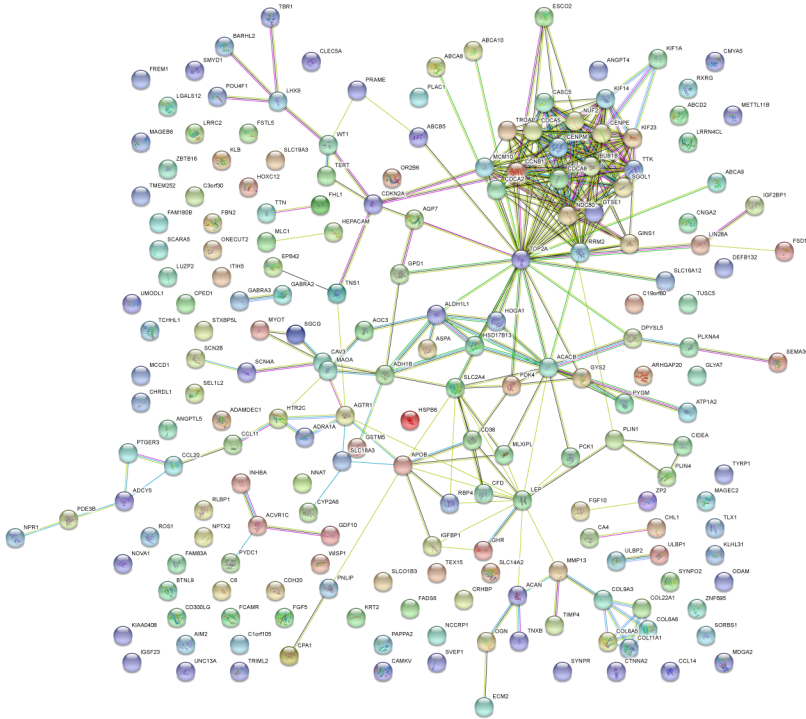

Supplement: Supplemental Information 2 — Each node represents a protein, and the edge between nodes represents the interaction between two connected proteins. [file peerj-07-7522-s002.pdf]

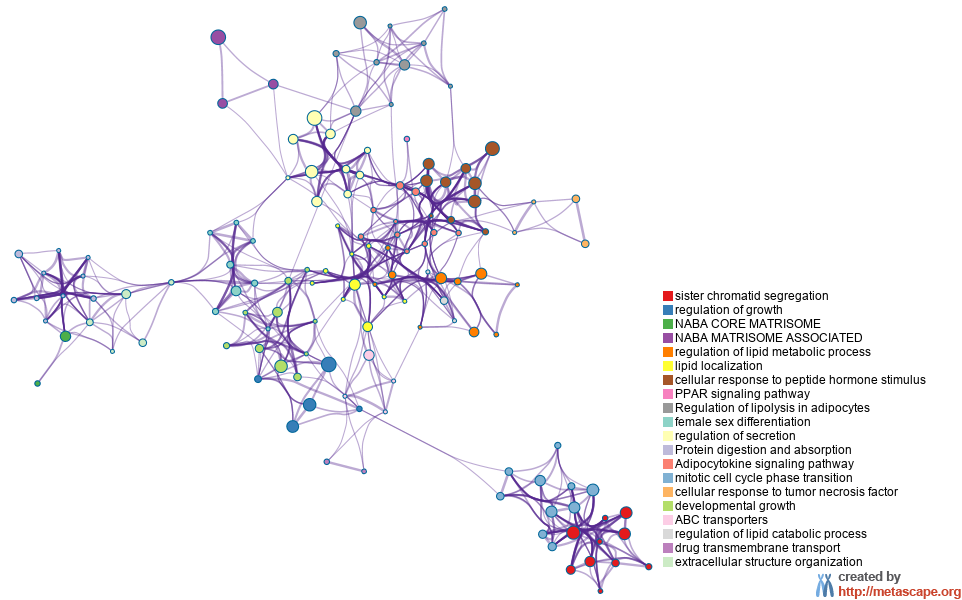

Supplement: Supplemental Information 4 [file peerj-07-7522-s004.zip › Supplemental Material 2/Enrichment_GO/ColorByCluster.png]

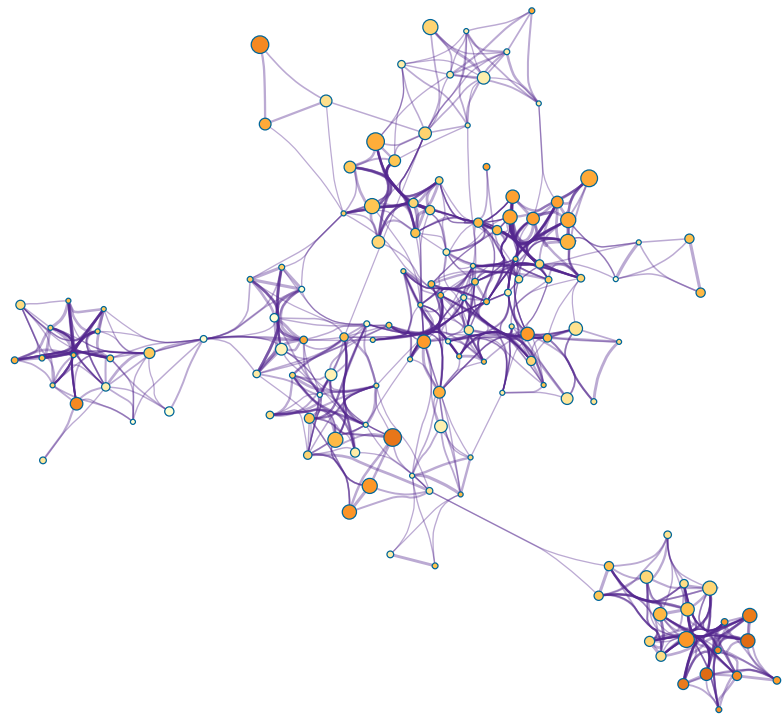

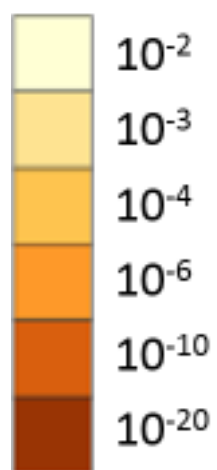

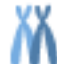 created by  
<http://metascape.org>

Supplement: Supplemental Information 4 [file peerj-07-7522-s004.zip › Supplemental Material 2/Enrichment_GO/ColorByPValue.pdf]

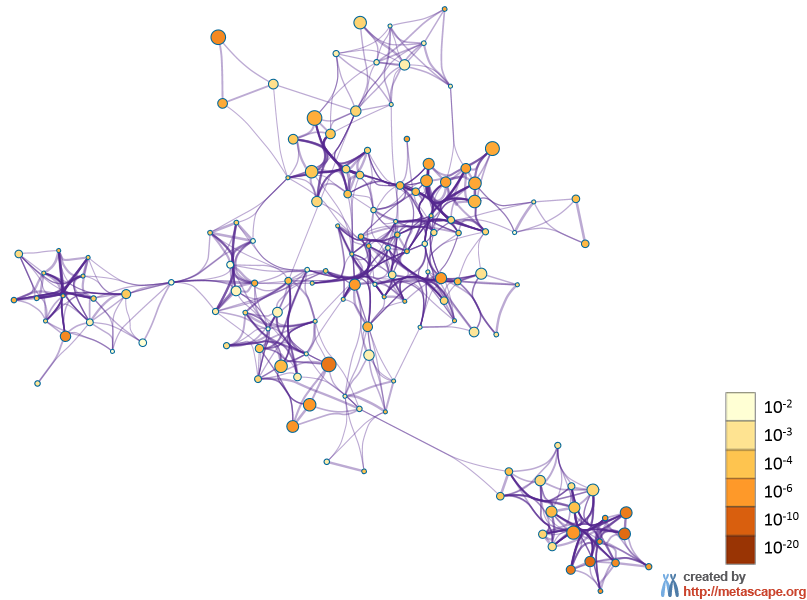

Supplement: Supplemental Information 4 [file peerj-07-7522-s004.zip › Supplemental Material 2/Enrichment_GO/ColorByPValue.png]

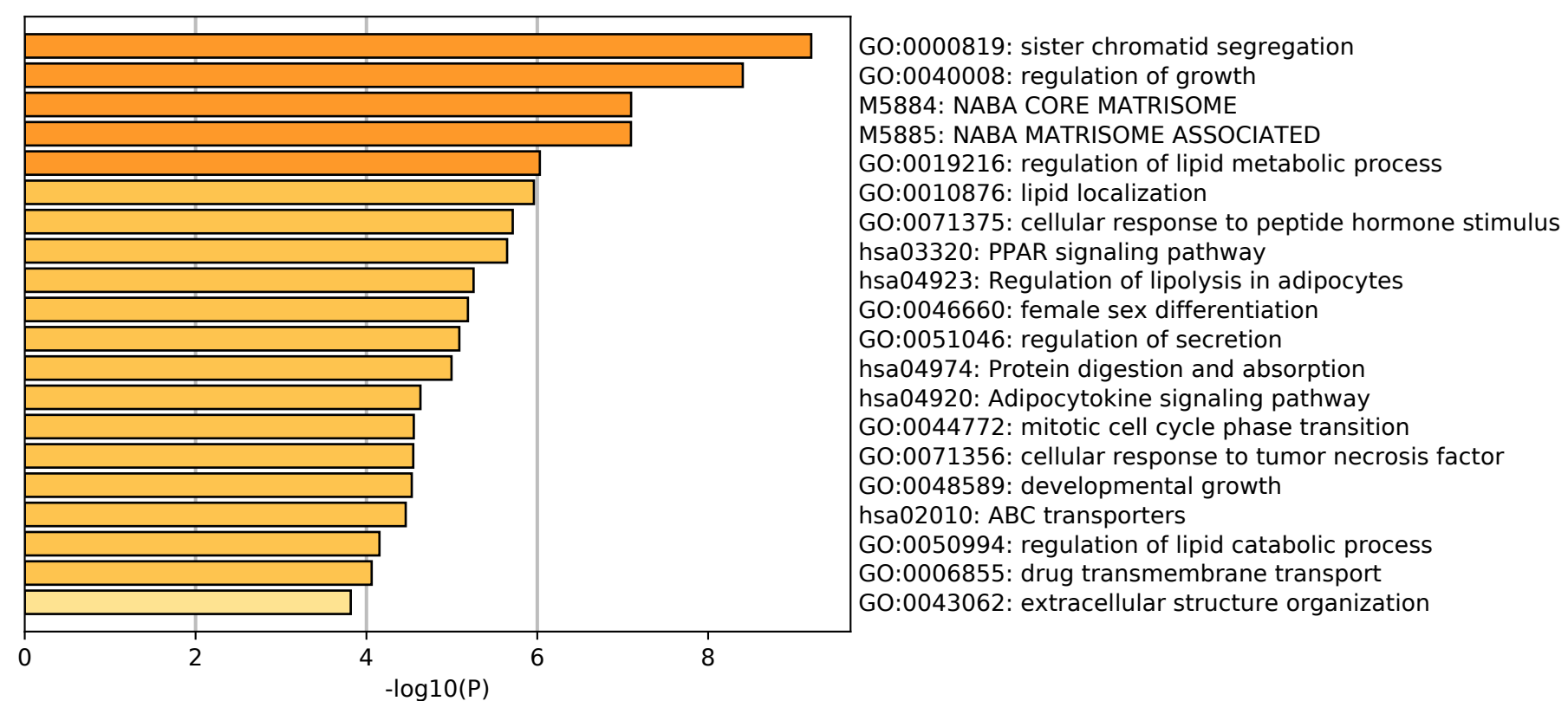

Supplement: Supplemental Information 4 [file peerj-07-7522-s004.zip › Supplemental Material 2/Enrichment_heatmap/HeatmapSelectedGO.pdf]

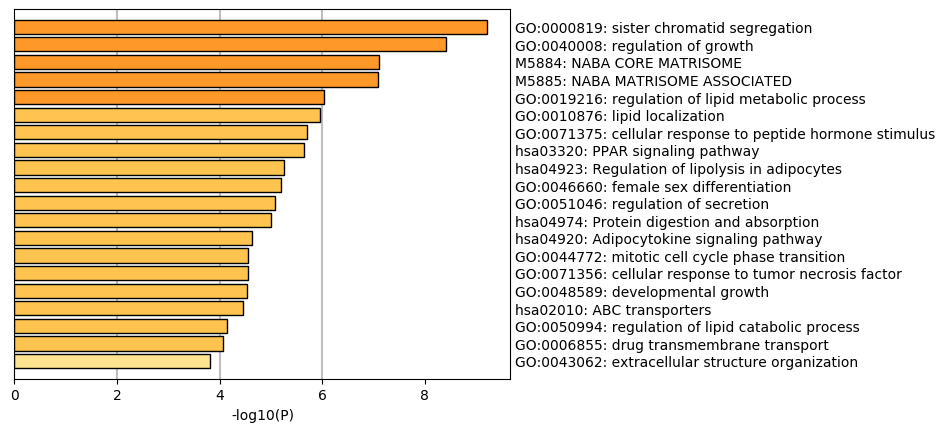

Supplement: Supplemental Information 4 [file peerj-07-7522-s004.zip › Supplemental Material 2/Enrichment_heatmap/HeatmapSelectedGO.png]
