# Supplementary material for: Characterization of ceRNA network to reveal potential prognostic biomarkers in triple-negative breast cancer
Source: PeerJ. 2019 Sep 9;7:e7522. doi: 10.7717/peerj.7522 (PMC6741283; doi:10.7717/peerj.7522)
Supplement: Supplemental Information 4 [file peerj-07-7522-s004.zip › Supplemental Material 2/Enrichment_GO/ColorByCluster.pdf]

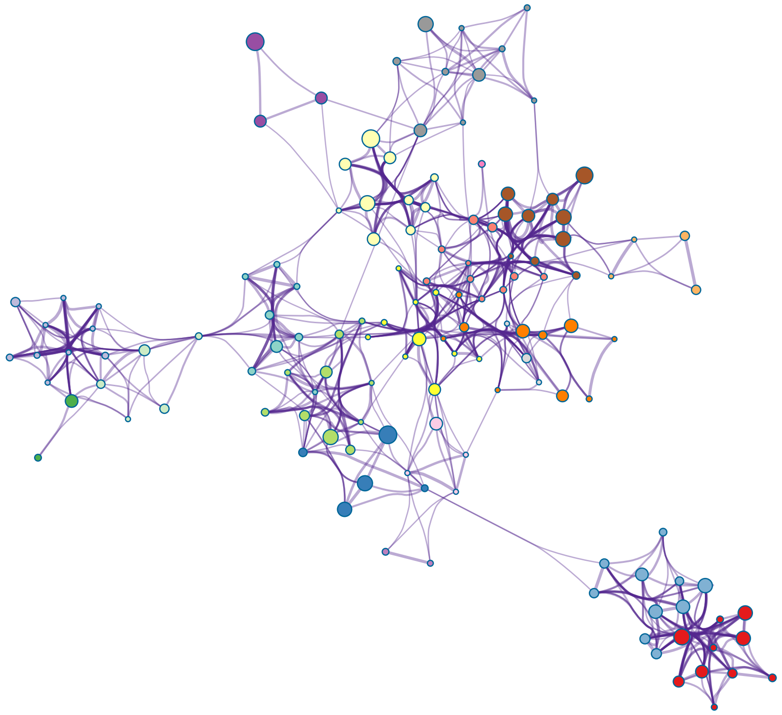

sister chromatid segregation
regulation of growth
NABA CORE MATRISOME
NABA MATRISOME ASSOCIATED
regulation of lipid metabolic process
lipid localization
cellular response to peptide hormone stimulus
PPAR signaling pathway
Regulation of lipolysis in adipocytes
female sex differentiation
regulation of secretion
Protein digestion and absorption
Adipocytokine signaling pathway
mitotic cell cycle phase transition
cellular response to tumor necrosis factor
developmental growth
ABC transporters
regulation of lipid catabolic process
drug transmembrane transport
extracellular structure organization

created by
http://metascape.org
