# Supplementary material for: Characterization of ceRNA network to reveal potential prognostic biomarkers in triple-negative breast cancer
Source: PeerJ. 2019 Sep 9;7:e7522. doi: 10.7717/peerj.7522 (PMC6741283; doi:10.7717/peerj.7522)
Supplement: Supplemental Information 5 [file peerj-07-7522-s005.zip › TableS1-S4/Table S1.docx]

Up-KEGG

|  |  |  |
| --- | --- | --- |
| **Term** | **P.value** | **Genes** |
| Systemic lupus erythematosus | 2.88277E-19 | HIST1H2AB, HIST1H2AG, HIST1H2AD, HIST2H4A, HIST1H2BO, HIST1H2BM, HIST1H2BK, HIST1H2BL, HIST1H4B, FCGR1A, HIST1H2BI, HIST2H2AC, HIST1H2BJ, IFNG, HIST1H4E, H2AFX, HIST3H2A, HIST1H4D, HIST3H2BB, HIST1H4I, HIST1H4H, HIST1H2BA, HIST1H3J, HIST1H2BE, HIST1H2BF, HIST1H2BH, CD80, HIST2H2BF, HIST1H3B, HIST1H2AI, HIST1H3C, HIST1H3D, HIST1H2AJ, HIST1H3F, HIST1H2AM, HIST1H3G, HIST1H2AL, HIST1H3H, HIST1H3I |
| Alcoholism | 1.97803E-16 | HIST1H2AB, HIST1H2AG, HIST1H2AD, HIST2H4A, HIST1H2BO, HIST1H2BM, HIST1H2BK, HIST1H4B, HIST1H2BL, GRIN2D, HIST2H2AC, HIST1H2BI, HIST1H2BJ, HIST1H4E, H2AFX, HIST3H2A, HIST1H4D, CALML5, GNG4, HIST3H2BB, HIST1H4I, HIST1H4H, HIST1H2BA, HIST1H3J, HIST1H2BE, HIST1H2BF, HIST1H2BH, GNGT1, NPY, HIST2H2BF, HIST1H3B, HIST1H2AI, HIST1H3C, HIST1H3D, HIST1H2AJ, HIST1H3F, HIST1H2AM, HIST1H3G, HIST1H2AL, HIST1H3H, HIST1H3I |
| Cell cycle | 1.52547E-12 | E2F1, E2F2, PKMYT1, TTK, CHEK1, PTTG1, CCNE2, CCNE1, CDC45, CDKN2A, BUB1, ORC6, ORC1, CCNA2, CDC7, CDC6, CDK1, CDC20, ESPL1, MCM2, CDC25C, MCM4, CDC25A, CDC25B, CCNB1, MAD2L1, CCNB2, PLK1, BUB1B, SMC1B |
| Viral carcinogenesis | 3.18874E-05 | CHEK1, HIST2H4A, HIST1H2BO, CCNE2, CCNE1, HIST1H2BM, CDKN2A, HIST1H2BK, HIST1H2BL, HIST1H4B, HIST1H2BI, HIST1H2BJ, HIST1H4E, HIST1H4D, HIST3H2BB, HIST1H4I, CCNA2, HIST1H4H, HIST1H2BA, CDK1, HIST1H2BE, HIST1H2BF, HIST1H2BH, CDC20, CCR8, HIST2H2BF |
| Transcriptional misregulation in cancer | 0.000274502 | HIST1H3J, ETV7, MMP9, CXCL8, PAX5, GZMB, HMGA2, MMP3, WT1, MYCN, FCGR1A, HIST1H3B, HIST1H3C, HIST1H3D, HIST1H3F, TLX3, HIST1H3G, TLX1, HIST1H3H, SSX1, HIST1H3I |
| Oocyte meiosis | 0.000337423 | CDK1, PKMYT1, CDC20, ESPL1, AURKA, PTTG1, CDC25C, SPDYC, CCNE2, CCNE1, MAD2L1, PLK1, FBXO43, BUB1, CALML5, SMC1B |
| Cytokine-cytokine receptor interaction | 0.001201828 | IL21R, CXCL9, CXCL8, CXCR3, CXCL11, CCL7, IL11, CXCL10, IL12RB2, CCL11, OSM, AMH, CCR8, TNFRSF9, CCL20, RELT, CXCR5, IFNB1, IFNG, TNFRSF18, IL12B, LTB, LTA, EPO |
| Progesterone-mediated oocyte maturation | 0.004049367 | CCNB1, CDK1, MAD2L1, CCNB2, PLK1, BUB1, PKMYT1, CDC25C, CCNA2, CDC25A, CDC25B, SPDYC |
| p53 signaling pathway | 0.006046258 | CCNB1, CCNE2, CCNE1, CDK1, CDKN2A, CCNB2, RRM2, RPRM, CHEK1, GTSE1 |
| Protein digestion and absorption | 0.012562576 | COL9A1, COL9A3, SLC15A1, COL6A5, KCNK5, COL22A1, PRSS1, ATP1A3, COL2A1, COL11A1, COL10A1 |
| Nicotine addiction | 0.014054989 | GABRD, GABRA3, GRIN2D, GABRA5, CHRNA6, GABRQ, CACNA1B |
| Bladder cancer | 0.015787777 | E2F1, E2F2, TYMP, CDKN2A, MMP9, CXCL8, MMP1 |
| ECM-receptor interaction | 0.030249008 | IBSP, SDC1, COL6A5, TNR, COMP, COL2A1, COL11A1, SPP1, FN1, HMMR |
| Neuroactive ligand-receptor interaction | 0.038618236 | GABRD, CCKBR, GABRA3, NPBWR1, OPRK1, GABRA5, PRSS1, GABBR2, NTSR1, GRM4, P2RY6, HRH3, GRM8, GRIN2D, P2RX2, HTR6, GALR2, CHRNA5, CHRNA6, HTR1D, HTR2C, GABRQ |
| Morphine addiction | 0.0388633 | GABRD, GNGT1, KCNJ6, GABRA3, GABRA5, GABBR2, GNG4, KCNJ3, GABRQ, CACNA1B |
| Tryptophan metabolism | 0.048761272 | TDO2, OGDHL, IDO2, IL4I1, IDO1, AOC1 |
| Fanconi anemia pathway | 0.048910705 | BLM, EME1, BRIP1, RMI2, FANCA, UBE2T, RAD51 |
|  |  |  |
