# Supplementary material for: Characterization of ceRNA network to reveal potential prognostic biomarkers in triple-negative breast cancer
Source: PeerJ. 2019 Sep 9;7:e7522. doi: 10.7717/peerj.7522 (PMC6741283; doi:10.7717/peerj.7522)
Supplement: Supplemental Information 5 [file peerj-07-7522-s005.zip › TableS1-S4/Table S2.docx]

KEGG down

| **Term** | **P,value** | **Genes** |
| --- | --- | --- |
| PPAR signaling pathway | 5.22588E-05 | LPL, SORBS1, PLIN1, PPARG, RXRG, FABP4, UCP1, ADIPOQ |
| Regulation of lipolysis in adipocytes | 0.001335868 | PTGER3, PLIN1, PDE3B, NPR1, FABP4, LIPE |
| Phenylalanine metabolism | 0.001826986 | GLYAT, MAOA, AOC2, AOC3 |
| AMPK signaling pathway | 0.002078118 | LEP, SLC2A4, LEPR, PFKFB1, PPARG, GYS2, ADIPOQ, LIPE |
| Drug metabolism - cytochrome P450 | 0.003171329 | GSTM2, FMO2, MAOA, CYP2A6, ADH1A, GSTM5 |
| Tyrosine metabolism | 0.014516295 | MAOA, ADH1A, AOC2, AOC3 |
| Glycine, serine and threonine metabolism | 0.019430159 | MAOA, DMGDH, AOC2, AOC3 |
| Adipocytokine signaling pathway | 0.019509677 | LEP, SLC2A4, LEPR, RXRG, ADIPOQ |
| Metabolism of xenobiotics by cytochrome P450 | 0.023419639 | GSTM2, CYP2A6, ADH1A, GSTM5, AKR1C1 |
| cAMP signaling pathway | 0.026684531 | FOS, PTGER3, PDE3B, NPR1, ATP1A2, HCAR3, HCAR1, LIPE |
