# Supplementary material for: Characterization of ceRNA network to reveal potential prognostic biomarkers in triple-negative breast cancer
Source: PeerJ. 2019 Sep 9;7:e7522. doi: 10.7717/peerj.7522 (PMC6741283; doi:10.7717/peerj.7522)
Supplement: Supplemental Information 5 [file peerj-07-7522-s005.zip › TableS1-S4/Table S3.docx]

GO analysis up

| Term | P.value | Genes |
| --- | --- | --- |
| nuclear division | 6.8954E-26 | KIFC1, XRCC2, PRC1, MAEL, PKMYT1, TTK, AURKA, PTTG1, AURKB, CDCA8, OIP5, CDCA2, CDCA5, CCNA2, ASPM, CDCA3, MND1, ESPL1, TACC3, MAD2L1, SPAG5, ZWINT, KIF4B, RAD21L1, KIF4A, NEK2, ASZ1, CHEK1, ANLN, MYBL2, SPC24, SPC25, FBXO43, SKA3, SKA1, ERCC6L, MKI67, EME1, NUF2, CDC20, NDC80, RAD54L, FAM64A, PLK1, SMC1B, KIF23, ZFP42, FAM83D, KIF2C, STRA8, FANCA, TOP2A, TUBB3, KIF14, CDC6, CDK1, KIF11, KIF15, DMRT1, TPX2, NUSAP1, PBK, HMGA2, UBE2C, RAD51, OSM, BUB1B, HORMAD1, UBE2S, BRSK2, CEP55, TDRD12, NCAPH, NCAPG, CENPA, BUB1, DAZL, TRIP13, CENPN, DLGAP5, PSRC1, KIF18A, KIF18B, CENPF, BIRC5, CENPE, RACGAP1, CDC25C, CDC25A, GSG2, CDC25B, FSD1, CCNB1, CCNB2, TEX15, CKS2, CENPW, CIT |
| cell cycle process | 1.4453E-24 | STIL, KIFC1, XRCC2, PRC1, MAEL, IQGAP3, PKMYT1, TTK, AURKA, PTTG1, AURKB, MCM10, AUNIP, EIF4EBP1, CDCA8, CDKN2A, OIP5, IFNG, CDCA2, ORC6, H2AFX, ORC1, CCNA2, CDCA5, ASPM, CDCA3, DTL, SIX3, MND1, ESPL1, TACC3, MAD2L1, SPAG5, ZWINT, STMN1, KIF4B, RAD21L1, KIF4A, BLM, TICRR, NEK2, SOX2, ASZ1, ANLN, CHEK1, MYBL2, SPC24, SPC25, FBXO43, SKA3, SKA1, DDIAS, TUBB4A, ERCC6L, GINS1, GINS2, MKI67, EME1, NUF2, BRIP1, CDC20, NDC80, DONSON, RAD54L, DNA2, PLK4, FAM64A, WDR62, PLK1, SMC1B, E2F1, KIF23, CLSPN, E2F7, EZH2, ZFP42, E2F8, RPRM, GTSE1, CDT1, CCNE2, FAM83D, CCNE1, KIF2C, CDC45, CXCR5, STRA8, POU4F1, FANCA, TOP2A, TERT, TUBB3, KIF14, CDC7, CDK1, CDC6, KIF11, SOX11, KIF15, DMRT1, TPX2, NUSAP1, PBK, MCM2, UBE2C, HMGA2, MCM4, RAD51, OSM, INHBA, RRM2, BUB1B, HORMAD1, IL12B, UBE2S, MELK, FOXM1, BRSK2, CXCL8, CEP55, TDRD12, HMMR, TYMS, NCAPH, NCAPG, CENPA, BUB1, GPSM2, DAZL, FEN1, TRIP13, FZD9, INSM1, CENPN, CENPM, DLGAP5, PSRC1, KIF18A, KIF18B, CENPF, BIRC5, CENPE, CDC25C, CDKN3, RACGAP1, CENPK, CDC25A, CENPI, GSG2, CDC25B, CCNB1, FSD1, CCNB2, TEX15, CKS2, CENPW, CENPU, CIT, FOXE3, KIF20A |
| organelle fission | 2.6275E-24 | KIFC1, XRCC2, PRC1, MAEL, PKMYT1, TTK, AURKA, PTTG1, AURKB, CDCA8, OIP5, CDCA2, CDCA5, CCNA2, ASPM, CDCA3, MND1, ESPL1, TACC3, MAD2L1, SPAG5, ZWINT, KIF4B, RAD21L1, KIF4A, NEK2, ASZ1, CHEK1, ANLN, MYBL2, SPC24, SPC25, FBXO43, SKA3, SKA1, ERCC6L, MKI67, EME1, NUF2, CDC20, NDC80, RAD54L, FAM64A, PLK1, SMC1B, KIF23, ZFP42, MTFR2, FAM83D, KIF2C, STRA8, FANCA, TOP2A, TUBB3, KIF14, CDC6, CDK1, KIF11, KIF15, DMRT1, TPX2, NUSAP1, PBK, HMGA2, UBE2C, RAD51, OSM, BUB1B, HORMAD1, UBE2S, BRSK2, CEP55, TDRD12, NCAPH, NCAPG, CENPA, BUB1, DAZL, TRIP13, CENPN, DLGAP5, PSRC1, KIF18A, KIF18B, CENPF, BIRC5, CENPE, RACGAP1, CDC25C, CDC25A, GSG2, CDC25B, FSD1, CCNB1, CCNB2, TEX15, CKS2, CENPW, CIT |
| DNA conformation change | 4.2812E-23 | HIST2H4A, HIST1H2BO, CDC45, HIST1H2BM, CDKN2A, HIST1H2BK, STRA8, OIP5, HIST1H2BL, HIST1H2BI, HIST1H2BJ, H2AFX, HIST3H2BB, TOP2A, CDCA5, CDK1, HIST1H1B, NUSAP1, MCM2, HMGA2, HMGA1, MCM4, RAD51, HIST1H1T, HIST2H2BF, HMGB3, BLM, NCAPH, NCAPG, HJURP, HIST1H4B, CENPA, HIST1H4E, HIST1H4D, HIST1H4I, ASF1B, HIST1H4H, GINS1, RECQL4, HIST1H2BA, HIST1H3J, GINS2, CENPN, CENPM, HIST1H2BE, HIST1H2BF, PIF1, HIST1H2BH, GINS4, BRIP1, CENPK, CENPI, CCNB1, DNA2, HIST1H3B, CENPW, HIST1H3C, HIST1H3D, CENPU, HIST1H3F, HIST1H3G, HIST1H3H, HIST1H3I |
| chromosome segregation | 1.0848E-22 | KIF23, KIFC1, PRC1, MAEL, TTK, PTTG1, AURKB, FAM83D, KIF2C, CDCA8, STRA8, OIP5, CDCA2, TOP2A, CDCA5, KIF14, CDC6, KIF11, NUSAP1, ESPL1, UBE2C, TACC3, ESCO2, MAD2L1, SPAG5, ZWINT, BUB1B, HORMAD1, KIF4B, KIF4A, RAD21L1, NEK2, CEP55, SPC24, SPC25, NCAPH, CENPA, HJURP, NCAPG, BUB1, SKA3, SKA1, FEN1, TRIP13, ERCC6L, CENPN, CENPM, DLGAP5, PSRC1, EME1, KIF18A, NUF2, CENPF, KIF18B, NDC80, CENPE, CDC20, BIRC5, CENPK, RACGAP1, GSG2, CENPI, CCNB1, TEX15, PLK1, CENPW, CENPU, SMC1B |
| mitotic cell cycle process | 1.4638E-22 | STIL, KIFC1, PRC1, IQGAP3, PKMYT1, TTK, AURKA, PTTG1, AURKB, MCM10, CDCA8, EIF4EBP1, CDKN2A, OIP5, CDCA2, ORC6, CCNA2, CDCA5, ORC1, ASPM, CDCA3, ESPL1, TACC3, MAD2L1, SPAG5, ZWINT, STMN1, KIF4B, KIF4A, RAD21L1, BLM, NEK2, TICRR, ANLN, CHEK1, MYBL2, SPC24, SPC25, FBXO43, SKA3, SKA1, TUBB4A, ERCC6L, GINS1, GINS2, EME1, NUF2, CDC20, NDC80, DNA2, PLK4, FAM64A, WDR62, PLK1, KIF23, E2F1, CLSPN, E2F7, EZH2, GTSE1, CDT1, FAM83D, CCNE2, KIF2C, CCNE1, CDC45, TOP2A, TERT, TUBB3, KIF14, CDC7, CDK1, CDC6, KIF11, KIF15, DMRT1, TPX2, NUSAP1, PBK, MCM2, UBE2C, HMGA2, MCM4, INHBA, RRM2, BUB1B, UBE2S, MELK, FOXM1, BRSK2, CEP55, HMMR, TYMS, NCAPH, CENPA, NCAPG, BUB1, GPSM2, CENPN, DLGAP5, PSRC1, KIF18A, KIF18B, CENPF, BIRC5, CENPE, CDC25C, CDKN3, RACGAP1, CDC25A, GSG2, CDC25B, CCNB1, FSD1, CCNB2, CKS2, CENPW, CIT, KIF20A |
| sister chromatid segregation | 2.1233E-22 | KIF23, KIFC1, PRC1, TTK, AURKB, KIF2C, CDCA8, STRA8, TOP2A, CDCA5, KIF14, CDC6, NUSAP1, ESPL1, TACC3, UBE2C, MAD2L1, SPAG5, ZWINT, BUB1B, HORMAD1, KIF4B, RAD21L1, KIF4A, NEK2, CEP55, SPC24, SPC25, NCAPH, NCAPG, CENPA, BUB1, SKA1, FEN1, ERCC6L, CENPN, CENPM, DLGAP5, PSRC1, NUF2, KIF18A, CENPF, KIF18B, NDC80, CENPE, BIRC5, CDC20, CENPK, RACGAP1, CENPI, GSG2, CCNB1, PLK1, CENPU, SMC1B |
| mitotic nuclear division | 5.2792E-22 | KIF23, KIFC1, PRC1, PKMYT1, TTK, AURKA, AURKB, PTTG1, FAM83D, KIF2C, CDCA8, OIP5, CDCA2, CDCA5, CCNA2, ASPM, TUBB3, CDCA3, KIF14, CDK1, CDC6, KIF11, KIF15, TPX2, DMRT1, NUSAP1, ESPL1, PBK, UBE2C, HMGA2, TACC3, MAD2L1, SPAG5, ZWINT, BUB1B, UBE2S, KIF4B, KIF4A, RAD21L1, NEK2, BRSK2, CHEK1, ANLN, CEP55, MYBL2, SPC24, SPC25, NCAPH, CENPA, NCAPG, BUB1, SKA3, SKA1, ERCC6L, CENPN, DLGAP5, PSRC1, KIF18A, NUF2, CENPF, KIF18B, NDC80, CENPE, CDC20, BIRC5, CDC25C, RACGAP1, GSG2, CDC25A, CDC25B, FSD1, CCNB1, FAM64A, CCNB2, PLK1, CENPW, CIT |
| mitotic cell cycle | 1.9862E-21 | STIL, KIFC1, XRCC2, PRC1, IQGAP3, PKMYT1, TTK, AURKA, PTTG1, AURKB, MCM10, CDCA8, EIF4EBP1, CDKN2A, OIP5, CDCA2, ORC6, CCNA2, CDCA5, ORC1, ASPM, CDCA3, ESPL1, TACC3, MAD2L1, SPAG5, ZWINT, STMN1, KIF4B, KIF4A, RAD21L1, BLM, NEK2, TICRR, ANLN, CHEK1, MYBL2, SPC24, SPC25, FBXO43, SKA3, SKA1, PPP2R2C, TUBB4A, ERCC6L, GINS1, GINS2, MKI67, EME1, NUF2, CDC20, NDC80, DNA2, PLK4, FAM64A, WDR62, PLK1, KIF23, E2F1, CLSPN, E2F7, EZH2, E2F8, GTSE1, CDT1, CCNE2, FAM83D, KIF2C, CCNE1, CDC45, TOP2A, TERT, TUBB3, KIF14, CDC7, CDK1, CDC6, KIF11, KIF15, DMRT1, TPX2, NUSAP1, PBK, MCM2, UBE2C, HMGA2, MCM4, INHBA, RRM2, BUB1B, UBE2S, MELK, FOXM1, BRSK2, CEP55, HMMR, TYMS, NCAPH, CENPA, NCAPG, BUB1, GPSM2, CENPN, DLGAP5, PSRC1, KIF18A, KIF18B, CENPF, BIRC5, CENPE, CDC25C, CDKN3, RACGAP1, CDC25A, GSG2, CDC25B, CCNB1, FSD1, CCNB2, CKS2, CENPW, CIT, KIF20A |
| nuclear chromosome segregation | 2.2236E-21 | KIF23, KIFC1, PRC1, MAEL, TTK, PTTG1, AURKB, FAM83D, KIF2C, CDCA8, STRA8, TOP2A, CDCA5, KIF14, CDC6, NUSAP1, ESPL1, TACC3, UBE2C, MAD2L1, SPAG5, ZWINT, BUB1B, HORMAD1, KIF4B, RAD21L1, KIF4A, NEK2, CEP55, SPC24, SPC25, NCAPH, CENPA, NCAPG, BUB1, SKA1, FEN1, TRIP13, ERCC6L, CENPN, CENPM, DLGAP5, EME1, PSRC1, NUF2, KIF18A, CENPF, KIF18B, NDC80, CENPE, CDC20, BIRC5, CENPK, RACGAP1, CENPI, GSG2, CCNB1, TEX15, PLK1, CENPU, SMC1B |
| DNA packaging | 4.5471E-21 | HIST2H4A, HIST1H2BO, NCAPH, HIST1H2BM, CDKN2A, STRA8, HIST1H2BK, CENPA, HIST1H4B, HIST1H2BL, HJURP, NCAPG, OIP5, HIST1H2BI, HIST1H2BJ, HIST1H4E, H2AFX, HIST1H4D, HIST1H4I, HIST3H2BB, ASF1B, TOP2A, CDCA5, HIST1H4H, HIST1H2BA, CDK1, HIST1H3J, CENPN, CENPM, HIST1H2BE, HIST1H1B, HIST1H2BF, HIST1H2BH, NUSAP1, MCM2, HMGA2, CENPK, HMGA1, CENPI, CCNB1, HIST1H1T, HIST2H2BF, HIST1H3B, HIST1H3C, CENPW, HIST1H3D, CENPU, HIST1H3F, HIST1H3G, HIST1H3H, HIST1H3I |
| chromosome organization | 1.2773E-20 | KIFC1, PRC1, MAEL, TTK, CBX2, AURKA, PTTG1, AURKB, HIST1H2BO, HIST1H2BM, CDCA8, CDKN2A, HIST1H2BK, HIST1H2BL, OIP5, HIST1H2BI, HIST1H2BJ, H2AFX, CDCA5, ESPL1, TACC3, UHRF1, MAD2L1, SPAG5, ZWINT, KIF4B, KIF4A, RAD21L1, HMGB3, BLM, NEK2, SOX2, CHEK1, SPC24, SPC25, HIST1H4B, HIST1H4E, HIST1H4D, SKA1, HIST1H4I, ASF1B, DNMT3B, ERCC6L, HIST1H4H, GINS1, RECQL4, HIST1H3J, GINS2, CPA4, MKI67, PIF1, GINS4, NUF2, BRIP1, ATAD2, CDC20, NDC80, FOXP3, RAD54L, DPPA2, DNA2, PLK1, HIST1H3B, HIST1H3C, HIST1H3D, HIST1H3F, HIST1H3G, HIST1H3H, HIST1H3I, SMC1B, KIF23, EZH2, PAX5, HIST2H4A, KIF2C, CDC45, STRA8, HIST2H2AC, HIST3H2A, HIST3H2BB, TOP2A, TERT, KIF14, CDK1, CDC6, HIST1H1B, PADI3, NUSAP1, MCM2, UBE2C, HMGA2, MCM4, HMGA1, PADI1, RAD51, HIST1H1T, HIST2H2BF, BUB1B, HORMAD1, HIST1H2AB, HIST1H2AG, HIST1H2AD, CEP55, PRDM13, NCAPH, CENPA, HJURP, NCAPG, BUB1, FEN1, TRIP13, HIST1H2BA, CENPN, CENPM, HIST1H2BE, HIST1H2BF, DLGAP5, PSRC1, HIST1H2BH, KIF18A, KIF18B, CENPF, BIRC5, CENPE, RACGAP1, CENPK, GSG2, CENPI, CCNB1, TEX15, CD19, MAPK15, HIST1H2AI, CENPW, CENPU, HIST1H2AJ, HIST1H2AM, HIST1H2AL |
| cell cycle | 6.5972E-19 | STIL, KIFC1, XRCC2, PRC1, MAEL, IQGAP3, PKMYT1, TTK, AURKA, PTTG1, AURKB, MCM10, AUNIP, EIF4EBP1, CDCA8, CDKN2A, OIP5, IFNG, CDCA2, ORC6, H2AFX, ORC1, CCNA2, CDCA5, ASPM, CDCA3, DTL, SIX3, MND1, ESPL1, TACC3, ESCO2, UHRF1, MAD2L1, SPAG5, ZWINT, STMN1, KIF4B, RAD21L1, KIF4A, CDK5R2, BLM, TICRR, NEK2, SOX2, ASZ1, ANLN, CHEK1, MYBL2, SPC24, SPC25, FBXO43, SKA3, SKA1, DDIAS, PPP2R2C, TUBB4A, ERCC6L, GINS1, GINS2, MKI67, EME1, NUF2, BRIP1, CDC20, NDC80, DONSON, RAD54L, DNA2, PLK4, FAM64A, WDR62, PLK1, SMC1B, E2F1, KIF23, CLSPN, E2F2, E2F7, EZH2, ZFP42, E2F8, RPRM, GTSE1, CDT1, CCNE2, FAM83D, CCNE1, KIF2C, CDC45, CXCR5, STRA8, POU4F1, FANCA, TOP2A, TERT, TUBB3, KIF14, CDC7, CDK1, CDC6, KIF11, SOX11, KIF15, TPX2, DMRT1, NUSAP1, PBK, MCM2, UBE2C, HMGA2, MCM4, SPDYC, RAD51, OSM, INHBA, RRM2, BUB1B, HORMAD1, IL12B, UBE2S, MELK, FOXM1, BRSK2, CXCL8, CEP55, TDRD12, HMMR, TYMS, NCAPH, NCAPG, HJURP, CENPA, BUB1, GPSM2, DAZL, FEN1, TRIP13, FZD9, INSM1, EXO1, CENPN, CENPM, DLGAP5, PSRC1, KIF18A, KIF18B, CENPF, BIRC5, CENPE, CDC25C, CDKN3, RACGAP1, CENPK, CDC25A, CENPI, GSG2, CDC25B, CCNB1, FSD1, CCNB2, TEX15, CKS2, CHTF18, CENPW, CENPU, CIT, FOXE3, KIF20A |
| chromatin assembly | 1.9606E-18 | HIST2H4A, HIST1H2BO, HIST1H2BM, CDKN2A, HIST1H2BK, CENPA, HIST1H4B, OIP5, HJURP, HIST1H2BL, HIST1H2BI, HIST1H2BJ, HIST1H4E, H2AFX, HIST1H4D, ASF1B, HIST3H2BB, HIST1H4I, HIST1H4H, HIST1H2BA, HIST1H3J, CENPN, CENPM, HIST1H2BE, HIST1H1B, HIST1H2BF, HIST1H2BH, MCM2, CENPK, HMGA2, HMGA1, CENPI, HIST1H1T, HIST2H2BF, HIST1H3B, HIST1H3C, CENPW, HIST1H3D, CENPU, HIST1H3F, HIST1H3G, HIST1H3H, HIST1H3I |
| cell division | 9.0796E-18 | KIF23, FGF5, KIFC1, PRC1, E2F7, E2F8, AURKA, PTTG1, AURKB, CCNE2, FAM83D, KIF2C, CCNE1, CDCA8, CDKN2A, CDCA7, STRA8, CXCR5, OIP5, CDCA2, TOP2A, CCNA2, CDCA5, ASPM, TUBA1C, CDCA3, KIF14, CDC7, CDK1, CDC6, KIF11, TPX2, NUSAP1, ESPL1, UBE2C, HMGA2, TACC3, OSM, MAD2L1, SPAG5, ZWINT, BUB1B, STMN1, UBE2S, KIF4B, KIF4A, BLM, NEK2, BRSK2, ANLN, CEP55, SPC24, SPC25, NCAPH, CENPA, NCAPG, BUB1, SKA3, POU3F2, SKA1, ERCC6L, PSRC1, NUF2, CENPF, KIF18B, NDC80, CENPE, CDC20, BIRC5, CDC25C, RACGAP1, CDC25A, CDC25B, FSD1, CCNB1, FAM64A, CCNB2, PLK1, CKS2, CENPW, CIT, WNT7A, KIF20A |
| nucleosome assembly | 1.21E-17 | HIST2H4A, HIST1H2BO, HIST1H2BM, HIST1H2BK, OIP5, HJURP, HIST1H2BL, HIST1H4B, CENPA, HIST1H2BI, HIST1H2BJ, HIST1H4E, H2AFX, HIST1H4D, ASF1B, HIST3H2BB, HIST1H4I, HIST1H4H, HIST1H2BA, HIST1H3J, CENPN, CENPM, HIST1H2BE, HIST1H1B, HIST1H2BF, HIST1H2BH, MCM2, CENPK, CENPI, HIST1H1T, HIST2H2BF, HIST1H3B, HIST1H3C, CENPW, HIST1H3D, CENPU, HIST1H3F, HIST1H3G, HIST1H3H, HIST1H3I |
| mitotic sister chromatid segregation | 6.045E-17 | KIF23, KIFC1, KIF4B, RAD21L1, KIF4A, PRC1, NEK2, TTK, CEP55, AURKB, KIF2C, CDCA8, NCAPH, CENPA, NCAPG, BUB1, CDCA5, KIF14, CDC6, DLGAP5, PSRC1, KIF18A, CENPF, KIF18B, NUSAP1, NDC80, ESPL1, CENPE, RACGAP1, TACC3, UBE2C, GSG2, CCNB1, MAD2L1, SPAG5, PLK1, ZWINT, BUB1B |
| chromatin assembly or disassembly | 6.379E-17 | HIST2H4A, HIST1H2BO, HIST1H2BM, CDKN2A, HIST1H2BK, CENPA, HIST1H4B, OIP5, HJURP, HIST1H2BL, HIST1H2BI, HIST1H2BJ, HIST1H4E, H2AFX, HIST3H2A, HIST1H4D, ASF1B, HIST3H2BB, HIST1H4I, HIST1H4H, HIST1H2BA, HIST1H3J, CENPN, CENPM, HIST1H2BE, HIST1H1B, HIST1H2BF, HIST1H2BH, MCM2, CENPK, HMGA2, HMGA1, CENPI, HIST1H1T, HIST2H2BF, HIST1H3B, HIST1H3C, CENPW, HIST1H3D, CENPU, HIST1H3F, HIST1H3G, HIST1H3H, HIST1H3I |
| nucleosome organization | 1.4369E-16 | HIST2H4A, HIST1H2BO, HIST1H2BM, HIST1H2BK, CENPA, OIP5, HJURP, HIST1H2BL, HIST1H4B, HIST1H2BI, HIST1H2BJ, HIST1H4E, H2AFX, HIST3H2A, HIST1H4D, ASF1B, HIST3H2BB, HIST1H4I, HIST1H4H, HIST1H2BA, HIST1H3J, CENPN, CENPM, HIST1H2BE, HIST1H1B, HIST1H2BF, HIST1H2BH, MCM2, CENPK, HMGA1, CENPI, HIST1H1T, HIST2H2BF, HIST1H3B, HIST1H3C, CENPW, HIST1H3D, CENPU, HIST1H3F, HIST1H3G, HIST1H3H, HIST1H3I |
| sister chromatid cohesion | 3.1646E-14 | RAD21L1, AURKB, SPC24, SPC25, KIF2C, CDCA8, STRA8, CENPA, BUB1, SKA1, CDCA5, FEN1, ERCC6L, CENPN, CENPM, KIF18A, NUF2, CENPF, NDC80, ESPL1, BIRC5, CDC20, CENPE, CENPK, CENPI, GSG2, MAD2L1, PLK1, ZWINT, BUB1B, HORMAD1, CENPU, SMC1B |
