# Supplementary material for: Characterization of ceRNA network to reveal potential prognostic biomarkers in triple-negative breast cancer
Source: PeerJ. 2019 Sep 9;7:e7522. doi: 10.7717/peerj.7522 (PMC6741283; doi:10.7717/peerj.7522)
Supplement: Supplemental Information 5 [file peerj-07-7522-s005.zip › TableS1-S4/Table S4.docx]

GO analysis down

| **Term** | **P**.**value** | **Genes** |
| --- | --- | --- |
| muscle system process | 1.51E-13 | CAV3, SCN3B, NPY2R, SRL, KCNIP2, TTN, MYOT, GSTM2, SORBS1, ANK2, KCNE1, CNN1, SCN7A, MYOM1, MYOC, CACNA2D1, PTGER3, MYH1, SCN2B, MYH2, KLF15, ATP1A2, CTNNA3, LEP, TNNT3, HSPB6, MYH11, KLF4 |
| muscle contraction | 2.29E-10 | CAV3, CACNA2D1, PTGER3, MYH1, SCN2B, SCN3B, NPY2R, MYH2, ATP1A2, TTN, KCNIP2, CTNNA3, MYOT, TNNT3, GSTM2, SORBS1, ANK2, KCNE1, MYH11, CNN1, MYOM1, SCN7A |
| regulation of system process | 8.12E-08 | CAV3, CACNA2D1, PTGER3, SCN2B, SCN3B, NPY2R, CRHBP, SRL, FGF10, NPR1, ATP1A2, KCNIP2, ADIPOQ, CTNNA3, ITPR1, LEP, TNNT3, GSTM2, HSPB6, ANK2, KCNE1, CNN1, KLF4 |
| membrane depolarization | 8.96E-08 | CAV3, CACNA2D1, ANK2, SCN2B, SCN3B, SCN9A, DCN, SCN7A, ATP1A2, ADIPOQ, MYOC |
| chemical homeostasis | 1.09E-07 | CAV3, SCN3B, FHL1, NPY2R, LEPR, PPARG, DCN, CXCL12, GSTM2, ANK2, SLC2A4, SAA1, SCN7A, MYOC, AKR1C1, LPL, CACNA2D1, PTGER3, STC2, SCN2B, EPB42, KL, RFX6, ACSM2A, NPR1, ATP1A2, ADIPOQ, ITPR1, LEP, SLC17A7, CYBRD1, FABP4, CMA1, GPAM |
| response to purine-containing compound | 1.84E-07 | P2RY12, GSTM2, FOS, DUSP1, PDE2A, CRHBP, PFKFB1, PPARG, KCNE1, PER1, FOSB, CDO1, ADIPOQ |
| response to organonitrogen compound | 3.58E-07 | STC2, KL, CRHBP, PFKFB1, PPARG, PDE3B, TIMP4, KLF15, FOSB, ATP1A2, CDO1, ADIPOQ, CXCL12, LEP, P2RY12, FOS, GSTM2, PDE2A, SLC2A4, SORBS1, DUSP1, NTRK2, KCNE1, PER1, GLRA4, IGFBP1, KLF4, MGARP |
| response to organophosphorus | 4.75E-07 | P2RY12, FOS, DUSP1, PDE2A, CRHBP, PFKFB1, KCNE1, PER1, FOSB, CDO1, ADIPOQ, AKR1C1 |
| response to oxygen-containing compound | 5.18E-07 | CRHBP, PFKFB1, CXCL2, PPARG, PDE3B, FGF10, TIMP4, DCN, CXCL12, ALDH1A2, FOS, SLC2A4, SORBS1, KCNE1, PER1, GYS2, MGARP, AKR1C1, ZFP36, LPL, STC2, KL, RFX6, KLF15, FOSB, UCP1, ATP1A2, CDO1, ADIPOQ, SLIT3, P2RY12, LEP, PDE2A, DUSP1, NTRK2, CMA1, GLRA4, IGFBP1, GPAM, KLF4 |
| cardiac muscle cell action potential | 7.47E-07 | CAV3, CACNA2D1, ANK2, SCN2B, SCN3B, KCNE1, ATP1A2, KCNIP2, CTNNA3 |
| membrane depolarization during action potential | 9.99E-07 | CAV3, CACNA2D1, ANK2, SCN2B, SCN3B, SCN9A, SCN7A, ATP1A2 |
| system process | 1.22E-06 | CAV3, SCN3B, CRHBP, NPY2R, SRL, PPARG, FGF10, KCNIP2, TTN, CXCL12, MYOT, SCTR, FOS, GSTM2, ANK2, SORBS1, KCNE1, SCN9A, CNN1, SCN7A, MYOM1, MYOC, AKR1C1, CACNA2D1, PTGER3, SCN2B, MYH1, TMC2, MYH2, NPR1, KLF15, ATP1A2, ADIPOQ, ITPR1, CTNNA3, SLC17A7, LEP, TNNT3, PDE2A, HSPB6, SGCG, CLIC5, NTRK2, MYH11, CMA1, AOC2, CHL1, KLF4 |
| cellular response to endogenous stimulus | 1.71E-06 | CAV3, LEPR, CRHBP, PPARG, PDE3B, FGF10, FOS, GSTM2, GPC3, SLC2A4, SORBS1, KCNE1, PER1, MGARP, AKR1C1, ZFP36, EGR3, KLB, KL, RXRG, KLF15, FOSB, UCP1, ATP1A2, ADIPOQ, SLIT3, P2RY12, LEP, CHRDL1, PDE2A, DUSP1, NTRK2, IGFBP1, KLF4 |
| membrane depolarization during cardiac muscle cell action potential | 1.92E-06 | CAV3, CACNA2D1, ANK2, SCN2B, SCN3B, ATP1A2 |
| response to endogenous stimulus | 2.01E-06 | CAV3, CRHBP, LEPR, PFKFB1, PPARG, PDE3B, FGF10, TIMP4, CXCL12, FOS, GSTM2, GPC3, SLC2A4, SORBS1, KCNE1, PER1, MGARP, AKR1C1, ZFP36, EGR3, KLB, STC2, KL, RXRG, KLF15, FOSB, UCP1, ATP1A2, CDO1, ADIPOQ, SLIT3, P2RY12, LEP, CHRDL1, PDE2A, DUSP1, NTRK2, GLRA4, IGFBP1, KLF4 |
| actin-mediated cell contraction | 2.21E-06 | CAV3, TNNT3, CACNA2D1, ANK2, SCN2B, SCN3B, MYH2, KCNE1, TTN, CTNNA3 |
| action potential | 2.9E-06 | CAV3, CACNA2D1, ANK2, SCN2B, SCN3B, KCNE1, SCN9A, SCN7A, ATP1A2, KCNIP2, CTNNA3 |
| actin filament-based movement | 2.9E-06 | CAV3, TNNT3, CACNA2D1, ANK2, SCN2B, SCN3B, MYH2, KCNE1, ATP1A2, TTN, CTNNA3 |
| ion transport | 3.29E-06 | CAV3, GRIK1, SLC22A12, SCN3B, FHL1, CRHBP, SRL, SLC16A12, PPARG, KCNIP2, CXCL12, TMEM37, GSTM2, ANK2, SLC1A7, SCN9A, KCNE1, PER1, SCN7A, AKR1C1, AHNAK, CACNA2D1, STC2, SCN2B, COX7A1, TMC2, SLC7A10, UCP1, ATP1A2, ITPR1, P2RY12, LEP, SLC17A7, PDE2A, CLIC5, NTRK2, CA4, GLRA4 |
| response to nitrogen compound | 3.98E-06 | STC2, KL, CRHBP, PFKFB1, PPARG, PDE3B, TIMP4, KLF15, FOSB, ATP1A2, CDO1, ADIPOQ, CXCL12, LEP, P2RY12, FOS, GSTM2, PDE2A, SLC2A4, SORBS1, DUSP1, NTRK2, KCNE1, PER1, GLRA4, IGFBP1, KLF4, MGARP |
